# Epidemic prevalence information on social networks can mediate emergent collective outcomes in voluntary vaccine schemes

**DOI:** 10.1371/journal.pcbi.1006977

**Published:** 2019-05-23

**Authors:** Anupama Sharma, Shakti N. Menon, V. Sasidevan, Sitabhra Sinha

**Affiliations:** 1 The Institute of Mathematical Sciences, CIT Campus, Taramani, Chennai, India; 2 Department of Physics, Cochin University of Science and Technology, Cochin, India; London School of Hygiene & Tropical Medicine, UNITED KINGDOM

## Abstract

The effectiveness of a mass vaccination program can engender its own undoing if individuals choose to not get vaccinated believing that they are already protected by herd immunity. This would appear to be the optimal decision for an individual, based on a strategic appraisal of her costs and benefits, even though she would be vulnerable during subsequent outbreaks if the majority of the population argues in this manner. We investigate how voluntary vaccination can nevertheless emerge in a social network of rational agents, who make informed decisions whether to be vaccinated, integrated with a model of epidemic dynamics. The information available to each agent includes the prevalence of the disease in their local network neighborhood and/or globally in the population, as well as the fraction of their neighbors that are protected against the disease. Crucially, the payoffs governing the decision of agents vary with disease prevalence, resulting in the vaccine uptake behavior changing in response to contagion spreading. The collective behavior of the agents responding to local prevalence can lead to a significant reduction in the final epidemic size, particularly for less contagious diseases having low basic reproduction number $R_{0}$. Near the epidemic threshold ($R_{0}\approx 1$) the use of local prevalence information can result in divergent responses in the final vaccine coverage. Our results suggest that heterogeneity in the risk perception resulting from the spatio-temporal evolution of an epidemic differentially affects agents’ payoffs, which is a critical determinant of the success of voluntary vaccination schemes.

## Author summary

A major factor underlying the success of voluntary vaccination schemes is the public perception about the costs and benefits associated with vaccines. Individuals may avoid vaccination if they perceive the risk of infection to be low compared to the potential hazards and inconveniences associated with getting vaccinated. However, in the course of an epidemic outbreak individuals may opt to vaccinate because of the associated higher risk perception. Modeling individual decision-making in the presence of an evolving epidemic using games, we show that spatial heterogeneity in the vaccine-uptake behavior emerges with the spread of disease on social networks. Our results highlight the crucial importance of the information source shaping an individual’s risk perception for achieving high vaccine coverage.

## Introduction

Immunization through the vaccination of populations has been estimated to annually prevent 2-3 million deaths from infectious diseases such as measles, diphtheria, pertussis and tetanus [1]. This number may rise substantially with the development of strategies to further increase global vaccine coverage [2]. Apart from conferring a long-term protection against the disease to the vaccinated individual, vaccination has an even more important community-level benefit. A sufficiently high vaccine coverage makes it difficult for the pathogen to find susceptible hosts, thereby conferring *herd immunity* to the whole population [3, 4]. Consequently, even those members of the community who are unable to get vaccinated, such as newborns and immune-suppressed individuals, are protected against the disease. In principle, any disease caused by a pathogen that only has human hosts can be eradicated by mass immunization, provided there is a sufficiently efficacious vaccine that is readily available. Such an outcome has been realized for smallpox [5, 6] and is expected to be achieved for polio [7, 8]. Conversely, the presence of a significant fraction of non-immunized individuals, which disrupts the population’s herd immunity, can result in the recurrent outbreaks of vaccine-preventable diseases such as measles, mumps and pertussis [9]. Elucidating the mechanisms that promote wider acceptance of vaccination in the population can therefore help explicate the reasons behind the failure of immunization programs.

One of the most important challenges in implementing an effective immunization program is to ensure that enough individuals agree to get vaccinated. This decision could be based on many factors such as an individual’s knowledge about the costs, including perceived side-effects, and benefits of vaccination, as well as the social, economic and cultural environment to which they belong [10, 11]. The lack of public confidence in the efficacy and/or safety of vaccines can give rise to vaccine hesitancy (i.e., delay or refusal to get vaccinated despite the availability of vaccine services) [12], and in extreme cases generate vaccine scares [13, 14]. Even in the absence of any bias against a vaccine as such, vaccine uptake in the population may vary over time with changing prevalence of the disease. Indeed, it is expected that individuals will be more likely to get themselves vaccinated when there is a higher risk of getting infected [15]. Conversely, low disease incidence may often lead to a significant drop in vaccine uptake, presumably because of the lower perceived risk of contracting the disease [16]. This suggests that when the threat of infection is high the individual has a strong incentive to get vaccinated, while at times of lower risk she may be tempted to avoid vaccination and free-ride on the herd immunity provided by immunized members of a population without bearing any cost herself. However, if everyone argues in this manner and avoids vaccination, it would leave the population completely exposed to invasion by the pathogen. This is essentially an instance of a *social dilemma* [17] that often arises in strategic interactions between rational individuals, who are trying to maximize the benefits accruing to them from their actions and those of others [18]. That is, while free-riding appears to be optimal from an individual’s perspective, it leads to a clearly undesirable collective outcome. This is one of the problems central to game theory, which therefore provides a natural framework for understanding the conditions under which a population of rational individuals will voluntarily decide to get vaccinated.

Most earlier studies of interaction between disease spreading and vaccine uptake behavior that incorporated a game theoretic framework have assumed homogeneous, well-mixed populations [19–22]. Thus, the risk of infection for every individual, as well as the protection offered to them by immunized individuals in their neighborhood, is identical. However, in reality, individuals interact primarily with neighboring members of their social networks and can have widely different contact structures [23]. Considering the network microstructure governing contacts between individuals can explain aspects of the collective outcomes of spreading contagion processes [24–31] and strategic interactions [32] that do not manifest in well-mixed models of populations. However, models that investigate vaccine uptake behavior by individuals in social networks typically do not incorporate strategic considerations in terms of explicit payoffs, i.e., the net benefit associated with specific collective actions. Instead, agents are assumed to imitate the behavior of their more “successful” neighbor [33, 34]. Additional model-based studies of voluntary vaccination by agents located on a social network have offered new perspectives on the impact of network contact structure [35], presence of local sub-groups [36], and the role of beliefs [37] and learning [38] on decision-making. As models incorporating strategic decision-making and those utilizing social network approaches each describe different aspects of vaccine uptake behavior (see [39] for a review), a framework combining both may come closer to capturing the complexity associated with such behavior in reality.

To understand the interaction between human behavior and epidemic dynamics [40–42], in this paper we present a model in which rational agents take strategic decisions to vaccinate themselves on the basis of information about the disease prevalence and the immune status of their neighbors on a social network. Each agent decides their action by playing a game against a hypothetical opponent who shares the same neighborhood as it. Unlike previous studies that use a similar framework of strategic interactions, in our model the payoffs defining the structure of the game incorporate real-time information on the specific situation prevailing in the network neighborhood and consequently vary dynamically amongst individuals. Thus, the games played by the different agents change over time with the spread of the disease across the network, resulting in an emergent spatio-temporal heterogeneity in the nature of the games. We find that this heterogeneity at the level of individual agents, in terms of both information available to them as well as their response, can have significant implications for population-level outcomes such as the final epidemic size and the extent of vaccine coverage. We also examine how the source of the information, viz., global (fraction of the population that is infected) or local (fraction of infected neighbors), that agents may use in assessing the risk of getting infected can lead to very different collective outcomes. The implications of our results reported here suggest that access to real-time information about the state of an evolving epidemic can change the risk perception and affect the vaccine uptake decisions taken by individuals. These in turn result in emergent patterns of collective choice behavior that may provide useful insights into the mechanisms driving vaccine acceptance, which could be relevant for public health planning.

## Model

In our model, we study the dynamics of two coupled processes, namely epidemic spreading and the evolution of vaccine uptake behavior, on a social network of *N* agents. The connection topology of the network is specified by the contact structure among individuals in a given population. The time-scale of an epidemic considered here is much shorter than durations over which the network structure may change significantly as a result of births, deaths and migrations of individuals. This makes it relevant in contexts where a population is suddenly confronted with a situation that warrants vaccination within a short time-frame, such as a scenario involving the accidental release of a pathogen for which a vaccine is available, or perhaps a bioterrorism incident [43, 44]. The spread of the disease over the network changes the status of an agent which, at any instant, can be in one of three possible states, namely, susceptible (*S*), infected (*I*), and recovered (*R*). We assume that recovery from the disease confers immunity from further infection to an agent. The disease is assumed to spread through direct contact between agents with a transmission rate *β*, while infected agents recover from the disease after an average time period of *τ*_*I*_. Thus, the disease dynamics follows the well-known SIR model [45]. We have explicitly verified that qualitatively similar results are obtained upon varying either *β* or *τ*_*I*_, keeping all other parameters fixed (see S1 Fig). Introducing *vaccination* in this framework allows a susceptible individual to avoid the possibility of getting infected by immediately achieving an immune status (which effectively corresponds to the *R* state).

As an epidemic propagates through the population, each agent can have access to *local information* about the number of infected cases among her network neighbors (i.e., with whom she has direct contact), as well as *global information* about the disease prevalence in the entire network. In reality, such information is obtained through different channels, e.g., via mass-media in the case of global information and through word of mouth for local information. The agents also have information about the extent to which their neighborhood offers them protection from the disease. This is provided by their knowledge of how many of their neighbors are immune as a result of either having recovered from the disease earlier, or through vaccination. Each agent utilizes the above information to determine their likelihood of getting infected. Based on this threat perception, the agents subsequently make a strategic decision on whether to get vaccinated by taking into account the “cost” associated with vaccination. This cost arises from the threat of side-effects, either real or perceived, as well as the effort involved in getting vaccinated, and tempts the agent to free-ride on the protection that may be offered by the immunity of their neighbors, particularly when the prevalence is low. By engaging in such behavior agents can enjoy the benefits of immunization without bearing the cost of getting vaccinated themselves. However, if every agent argues along the same lines, it will lead to extremely low vaccine uptake, causing the loss of herd immunity and exposing the population to the risk of an epidemic outbreak of a vaccine-preventable disease. This results in a dilemma for a population of well-informed rational agents, who decide their actions entirely on the basis of maximizing their individual payoffs.

As a game-theoretic framework provides a natural setting for investigating such social dilemmas, we model the vaccine uptake decision process of individual agents in terms of games. In order to make a strategic decision each agent plays a symmetric 2-person game against a virtual opponent who shares the same neighborhood and hence has identical information. Note that in the heterogeneous setting that we consider where the network neighborhood of each agent is distinct, the information on the basis of which she takes a decision also differs from agent to agent. Thus, each agent asks whether by changing her action she could have increased her payoff given her unique situation. In order to achieve this, we allow the focal agent to consider a virtual opponent to which she attributes information identical to that which she possesses, and follows the same decision process as herself. In other words, the agent plays against her virtual self in order to see if she could have done better had she chosen a different action with the same information and in the same setting.

**Fig 1:**
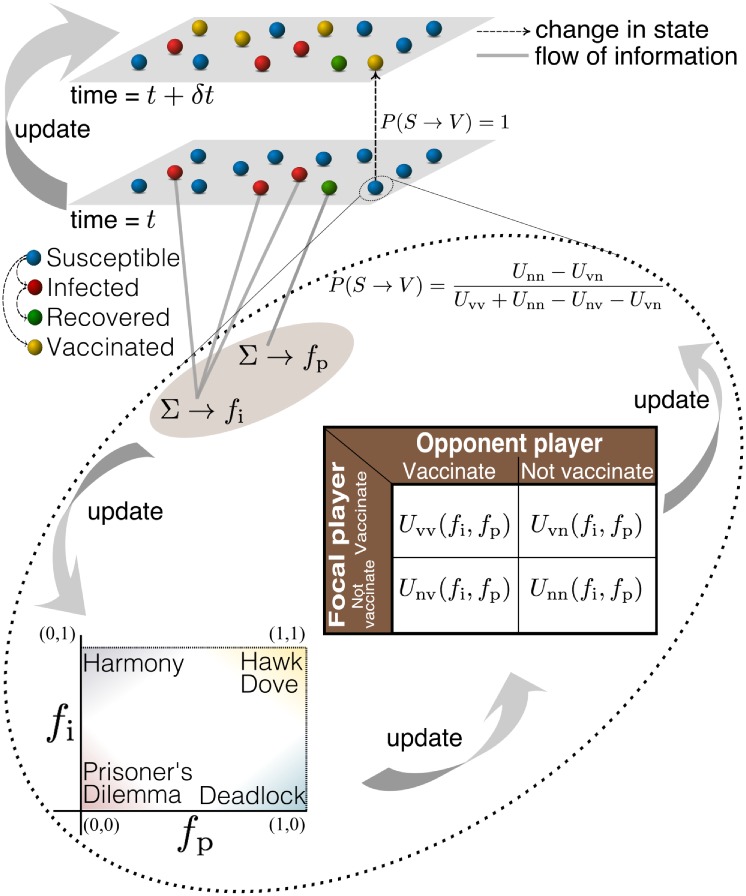
Schematic representation of the coupling between the spread of an epidemic and strategic vaccine uptake behavior by individuals. Agents are classified according to their state with respect the disease as Susceptible (S), Infected (I), Recovered (R) and Vaccinated (V). The two layers represent the states of the nodes at two time instants. The broken lines represent the change in the state of agents and grey solid lines represent the flow of information about the state (infected and removed) of agents in the network. The curved arrow between the two layers represents the update (time evolution) of the system. The broken curve encloses the game-theoretic process that determines whether an agent decides to vaccinate or not, based on the probability of an agent choosing to get vaccinated *P*(*S* → *V*). The table inside the broken curve is a payoff matrix used by an agent to make decisions. Here the “opponent” is a hypothetical agent having identical information, choices of actions and associated payoffs. The payoff received by the focal player is represented by a function of the form *U_xy_*(*f*_i_, *f*_p_), where x and y are the actions of the focal player and opponent respectively. The fractions of infected and protected (immune) agents are represented by *f*_i_ and *f*_p_, respectively. By varying these two parameters the nature of the game can change between different classes, as shown in the inset to the lower left.

At each round of the game, an agent has a choice of two possible actions, i.e., to get vaccinated (v) or not (n). The cost and benefit associated with the choices is represented in terms of a payoff matrix. An important feature of our approach is that the payoffs evolve with the progress of the epidemic and the ensuing change in vaccine coverage in the population. The payoff received by the focal player *j*, where *j* ∈ [1, *N*], is represented by a function of the form *U_xy_*(*f*_i_, *f*_p_), where x, y ∈ {n, v} are the actions of the focal player and the virtual opponent, respectively (see table in Fig 1). Here, *f*_p_ is the fraction of neighbors that are immune and *f*_i_ is a linear combination of local and global information about the disease prevalence:

$$\begin{array}{c} f_{i}\left(j\right)=\underset{\text{global}}{\underset{︸}{\alpha (I/N)}}+\underset{\text{local}}{\underset{︸}{\left(1-\alpha \right)(k_{i}\left(j\right)/k\left(j\right))}}. \end{array}$$

Note that *I* is the number of infected agents in a population of size *N*, while *k*(*j*) is the total number of neighbors of the focal agent *j*, of which *k*_i_(*j*) individuals are infected. By tuning the parameter *α* ∈ [0, 1], we can consider any information scenario between the two extreme cases wherein an agent uses exclusively local (*α* = 0) or global (*α* = 1) information.

As mentioned earlier, a high disease prevalence (i.e., a large value of *f*_i_) ensures that the benefits of vaccination outweigh its cost, thereby acting as an incentive for the focal agent to get vaccinated. It is reasonable to assume that the values of the payoffs associated with the decision to vaccinate increase with prevalence as, all other things remaining same, susceptible agents will be more likely to get infected when *f*_i_ is high. Specifically, it is beneficial to get vaccinated if even one of the neighbors of the focal agent remains susceptible to the disease. Thus the value of *f*_p_ will have less relevance in such a situation. Therefore, a reasonable simplification is to assume that *U*_vv_ and *U*_vn_ are increasing functions of *f*_i_ and independent of *f*_p_.

Another important consideration is when all the neighbors of the focal agent are immune to the disease. In this situation, there is a high probability that the agent will successfully escape infection even if she opts not to get vaccinated. Hence, analogous to the arguments used above, it is reasonable to assume that the payoffs associated with the decision to “not vaccinate” increase with the fraction of protected neighbors. In view of the fact that the utility of getting vaccinated depends primarily on the number of neighbors not protected against the disease, which is directly related to the probability of the focal agent to get infected, we consider *U*_nv_ and *U*_nn_ as increasing functions of *f*_p_ and independent of *f*_i_, as a simplification. For concreteness, we choose the simplest possible linear functional form for *U*_nv_, *U*_nn_, *U*_vv_ and *U*_vn_ as follows:

$$\begin{array}{ccc} & U_{\text{nv}}=af_{\text{p}}+b, & \;U_{\text{nn}}=cf_{\text{p}}+d,\\ & U_{\text{vn}}=ef_{i}+f, & \;U_{\text{vv}}=gf_{i}+h. \end{array}$$

This linear form in *f*_i_ and *f*_p_ has the added advantage of not having multiple solutions (i.e., Nash equilibria, explained later) for any particular choice of *f*_i_ and *f*_p_, which would have required invoking additional selection criteria for choosing among them. As the payoff functions are time-varying, the nature of the game can change depending on the hierarchical relation between the payoffs that prevails at any instant.

To characterize the hierarchy of payoff functions in the (*f*_i_, *f*_p_) space, we note that when *f*_i_ is high and *f*_p_ → 1, it is possible to escape infection as long as most of the neighbors are immune but in the absence of protection from the neighborhood, vaccination is vital to an individual. This suggests the following relation between payoffs: *U*_nv_ > *U*_vv_ > *U*_vn_ > *U*_nn_, i.e., the game is Hawk-Dove [46]. When *f*_i_ is low and *f*_p_ → 1, the non-vaccinators prevail as there a very low risk of infection and most of the population is immune to the disease. This would result in *U*_nv_ > *U*_nn_ > *U*_vv_ > *U*_vn_, i.e., the game is Deadlock [47]. When *f*_i_ is high and *f*_p_ → 0, the benefits of vaccination outweigh the perceived cost of vaccination because of the high risk of contracting disease. This results in the hierarchal relation *U*_vv_ > *U*_vn_ > *U*_nv_ > *U*_nn_, i.e., the game is Harmony [48]. When *f*_i_ is low and *f*_p_ → 0, it is extremely tempting to not get vaccinated because of low prevalence. However the possibility of being infected is non-zero, which makes vaccination a viable choice. This results in *U*_nv_ > *U*_vv_ > *U*_nn_ > *U*_vn_, i.e., the game is Prisoner’s Dilemma [49] (see Fig 1, inset). These four games govern the preference that an agent has for each action (viz., to vaccinate or to not vaccinate) at the four extremities of the (*f*_i_, *f*_p_) parameter space. In the interior of this space, the hierarchies among the payoffs gradually change, thereby giving rise to different games. To ascertain that the system behaves in the same way as explained above at these four extremities, we choose the parameters *a* − *h* such that *U*_nv_, *U*_nn_, *U*_vn_ and *U*_vv_ satisfies the inequalities mentioned above. The payoff associated with not getting vaccinated when the opponent chooses to vaccinate (*U*_nv_) is always greater than the corresponding payoff for the case where both do not get vaccinated (*U*_nn_), as the latter situation exposes both to the risk of being infected. We hence set *a* = *c* without loss of generality. Similarly, the payoff received when both the focal player and her virtual opponent get vaccinated (*U*_vv_) is greater than that obtained when only the focal player is vaccinated (*U*_vn_). This is because, in principle, the latter scenario implies that she, instead of her virtual opponent, could have avoided the cost associated with vaccination. We hence set *e* = *g* without loss of generality. If the parameters *a* − *h* satisfy the following relations:

$$\begin{array}{c} a+b>e+h>e+f>b,a+d>h>d>f, \end{array}$$ (1)

then the situations discussed above (Hawk-Dove, Deadlock, Harmony and Prisoners’ Dilemma) will prevail at the four extremities of the (*f*_i_, *f*_p_) space. As the information on the basis of which the agent decides whether to vaccinate changes over time, the nature of the game played by her also varies. Thus, while under certain conditions, agents can exhibit a propensity to free-ride (e.g., when prevalence is low), our model is also consistent with recent observations about the prevalence-elasticity of the demand for vaccines [50–52].

As the epidemic spreads in the population each susceptible agent *j* will, at any time *t*, choose an action such that a unilateral change of action will not yield a higher payoff. In game theory, such an action profile is known as a Nash equilibrium [53]. If player *j* (and her opponent) decides to vaccinate with probability *p*_*j*_ (*p*_*o*_) and not vaccinate with probability 1 − *p*_*j*_ (1 − *p*_*o*_), the expected utility for agent *j* can then be calculated as

$$\begin{array}{c} \epsilon_{j}=p_{j}(p_{o}(U_{\text{vv}}+U_{\text{nn}}-U_{\text{nv}}-U_{\text{vn}})+U_{\text{vn}}-U_{\text{nn}})+p_{o}\left(U_{\text{vn}}-U_{\text{nn}}\right)+U_{\text{nn}}. \end{array}$$

Given that the game is symmetric, the Nash equilibrium would be either *p*_*j*_ = 0 or *p*_*j*_ = 1 if it is pure, or if it is mixed then the agent *j* would vaccinate with the probability

$$\begin{array}{c} p_{j}=\frac{U_{\text{nn}}-U_{\text{vn}}}{U_{\text{vv}}+U_{\text{nn}}-U_{\text{nv}}-U_{\text{vn}}}. \end{array}$$

Note that the expression for the vaccination probability for a mixed strategy Nash equilibrium is similar to the strategy referred as mixed ESS in the Bishop-Cannings theorem [54]. As this probability will be different for each susceptible agent, it introduces heterogeneity in the individuals’ decision across the network due to differences in the risk-perception of each agent. Also, as this probability can change with time, an agent can change her decision as the disease spreads over the network. Incorporating such spatio-temporally varying strategies for the vaccine uptake of agents on a network presents a more realistic way of examining the coupled dynamics of vaccination and disease.

In order to study the consequences of the interplay between the strategic decision-making process for vaccine uptake and epidemic spreading, we simulate the stochastic spread of a directly transmitted disease on empirical social networks of villages in southern India [55], as well as model networks (the simulation algorithm is outlined in S1 Text). All agents in our model are initially susceptible and 0.5% of the nodes in a network are randomly chosen to become infected to simulate the onset of an epidemic. Note that no node is initially in a vaccinated state. We employ the Gillespie stochastic evolution algorithm [56] to determine the time at which the next event will happen and which node would take part in that event. The event could be one of the three different types of transitions that can change the state of a node: (i) disease transmission (*S* → *I*), (ii) recovery (*I* → *R*), and (iii) vaccination (*S* → *R*). Disease transmission is a contact-dependent transition and can take place only when node *j* in state *S* is in contact (i.e., has a connecting link) with nodes in state *I*. Recovery is a time-dependent transition and depends on the time interval spent by a node *j* in infected state (for more details see [57]). Vaccination is an information-driven transition, which involves strategic decision making (as shown in Fig 1). The simulation is stopped when there are no infected nodes remaining in the network. The payoff parameter values used for all simulations reported here are *a* = 0.45, *b* = 0.3, *d* = 0.002, *e* = 0.5, *f* = 0 and *h* = 0.2, which satisfy the relations (1). As shown in S2 Fig, the results reported here are robust with respect to different choices of these parameter values (which are in any case highly constrained by the above-mentioned inequalities).

## Results

**Fig 2:**
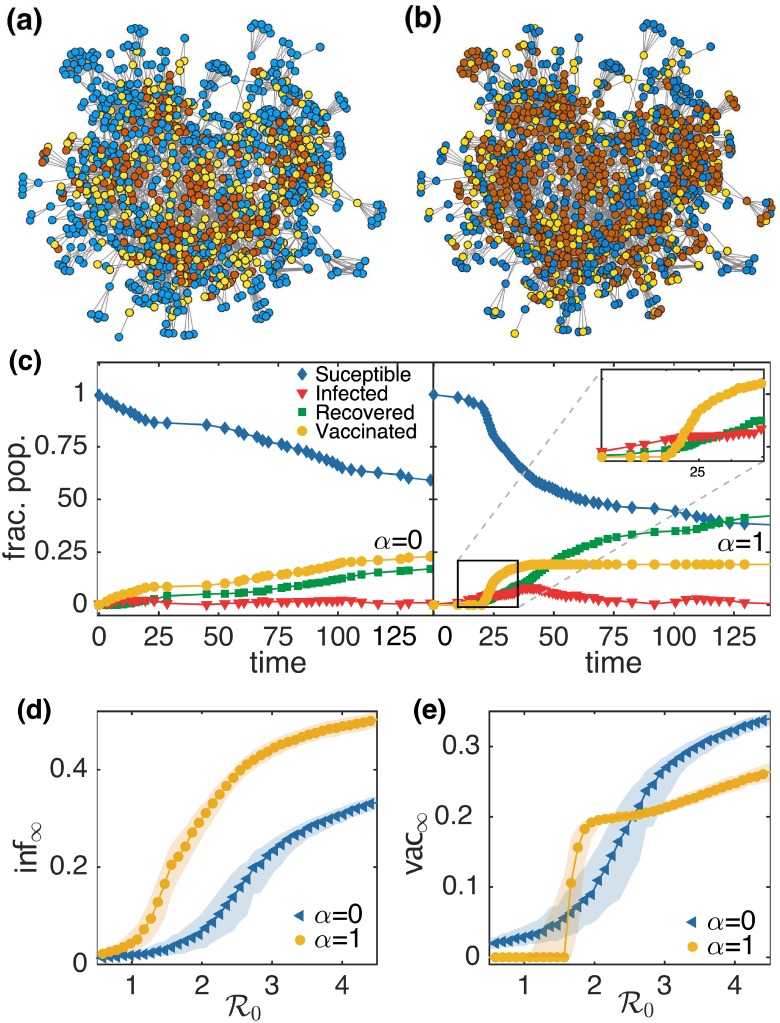
Simulation results for the co-evolution of epidemic spreading and vaccine uptake behavior in the largest connected component of a social network in a village of southern India. A snapshot of the network for village 55 (see Ref. [57], data obtained from Ref. [55]) with *N* = 1180 and 〈*k*〉 = 9.78, showing the final states of nodes following a simulated epidemic with *β* = 0.025 and *τ*_*I*_ = 10 for (a) *α* = 0 and (b) *α* = 1. The colors of the nodes are representative of the final state: blue, susceptible; yellow, vaccinated; red, recovered (i.e., infected during the epidemic). (c) A sample time series showing the evolution of *S*, *I*, *R* and *V* for a simulated epidemic with *β* = 0.025 and *τ*_*I*_ = 10 for *α* = 0 (left) and *α* = 1 (right). The inset of (c) provides a closer view of the sudden emergence of vaccination when the prevalence becomes sufficiently high. A comparison of the final fraction of agents (d) infected inf_∞_ and (e) vaccinated vac_∞_ during a simulated epidemic with different values of $R_{0}$, for *α* = 0 and *α* = 1. Each of the points represents the median of 1000 simulation runs and the patches indicate the interquartile range (IQR).

The goal of our study is to see if voluntary vaccination can emerge as a result of spatially heterogeneous strategic decision making in response to individual-based assessment of an epidemic threat and if so, what role the source of information (local or global) may play in shaping this collective response. Fig 2 shows the results obtained for a simulated epidemic on the social network of one of the 75 villages in southern India from the data set of [55]. We stress, however that our results are qualitatively similar for other choices of social network (as shown in the subsequent figure). Fig 2(a) and 2(b) illustrates the final outcome of a simulated epidemic with transmission rate *β* = 0.025 and average infectious period *τ*_*I*_ = 10 on the empirical social network of a specific village (village 55 in the data set), for the two extreme values of *α* (results for intermediate values of *α* are shown in S3 Fig). The blue color represents the nodes that escaped infection without getting vaccinated. Note that as all nodes were initially susceptible, the vaccine uptake behavior is entirely epidemic-driven. It is evident from the figure that more agents experience the disease (as indicated by red colored nodes) when the information available about prevalence is global (*α* = 1) as compared to when it is local (*α* = 0), although the vaccine coverage (as indicated by yellow colored nodes) is almost same. To understand the reason behind this disparity in the final outcome of epidemic simulated when considering different sources of information, we consider the time evolution of the fraction of nodes in different states, as shown in Fig 2(c). For *α* = 0, the final fraction of agents that were infected during the epidemic, inf_∞_, is 0.17 and the final fraction of agents vaccinated during the epidemic, vac_∞_, is 0.22. In contrast, for the case *α* = 1, inf_∞_ is 0.42 and vac_∞_ is 0.19. Hence, even though vac_∞_ is similar for the two cases, there is a significant difference in the value of inf_∞_. It is clear from the figure that voluntary vaccination behavior emerges much later in the case *α* = 1 (at *t* = 20) as compared to *α* = 0, where it emerges almost immediately after initiating the simulated epidemic. As highlighted in the inset of the right panel of Fig 2(c), in the case *α* = 1 the agents start getting vaccinated when the epidemic prevalence becomes significantly high. This emergent behavior is a reasonable description of how responses to epidemics typically unfold. For instance, in the absence of an efficient mechanism for the dissemination of incidence data, the media usually reports an outbreak only when the reported cases of the disease becomes sufficiently high. Once a disease has affected a significant proportion of population, even a subsequent high vaccine coverage would be unable to reduce the final fraction of infected agents. To test the robustness of these results with regard to the contagiousness of the epidemic, we simulated epidemics with different values of the basic reproduction number $R_{0}$, the average number of secondary cases resulting from a single primary infection in a completely susceptible population. For each value of $R_{0}$ we conduct 1000 trials to average over the effect of noise on the final size of the epidemic and vaccine coverage. On comparing the final outcome of these simulations, it is apparent that the value of inf_∞_ for *α* = 1 is always greater than the corresponding value for *α* = 0, independent of any choice of $R_{0}$ (Fig 2(d)). This underpins the previous observation that the epidemic infects a larger proportion of agents in the network when agents decide to get vaccinated based on the information about the global disease prevalence, as compared to local. However, a comparison of vac_∞_ for *α* = 0 and *α* = 1 reveals a more complex situation (see Fig 2e). For both low and high values of $R_{0}$, vac_∞_ is higher for *α* = 0 than for *α* = 1, but there is an intermediate range of $R_{0}$ in which the values of vac_∞_ for *α* = 1 are higher than for *α* = 0. Thus, there is a crossover of both the curves of vac_∞_ for *α* = 0 and *α* = 1. This shows that an epidemic simulated with these intermediate values of $R_{0}$ results in higher vaccine coverage when agents base their vaccination coverage on the global information as compared to local. An important point to note here is that the effect of high vaccine coverage in this regime of $R_{0}$ for *α* = 1 is not reflected in the final size of the epidemic (Fig 2(d)). This shows that even if the vaccine coverage in this regime of $R_{0}$ is high, the simulated epidemic affects more agents for *α* = 1 than for the case *α* = 0. A possible explanation of this is that in the case of global information the threat perception does not appear significant unless a large proportion of agents are affected by the epidemic and hence fails to overcome the perceived cost of vaccination. This results in limited vaccine uptake which does not provide any significant check on the spread of the epidemic. Furthermore, use of local prevalence information leads to localized elevated vaccine uptake in the neighborhoods of infectious agents which allow for efficient intervention. By contrast, vaccine usage is dispersed throughout the network in the case where global prevalence information is used, resulting in sub-optimal outcomes.

**Fig 3:**
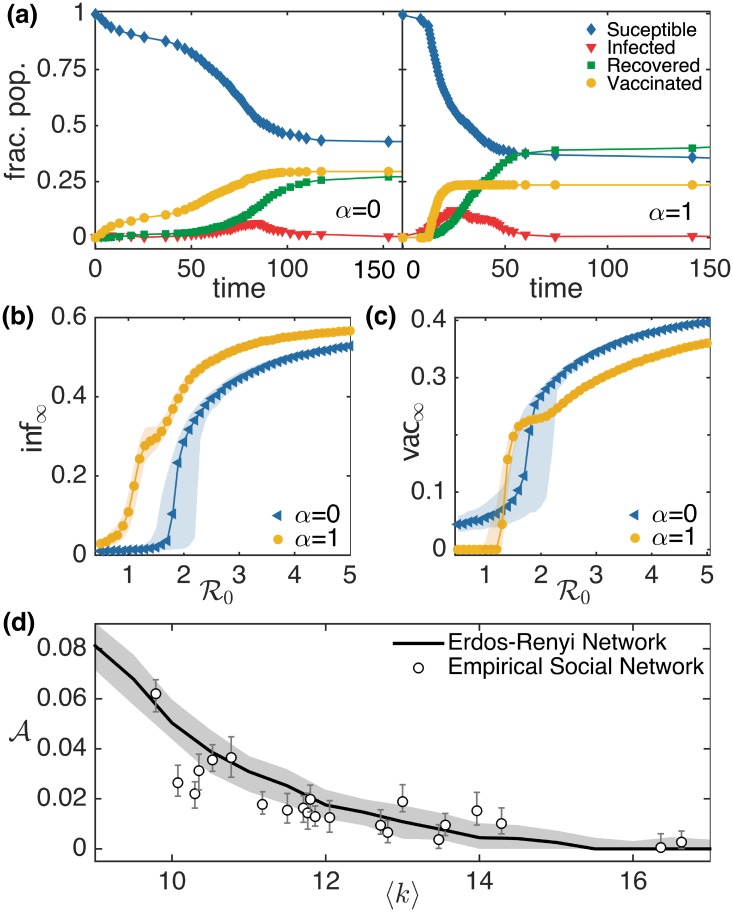
Simulation results for the co-evolution of epidemic spreading and vaccine uptake behavior in Erdős-Rényi networks. (a) A sample time series showing the evolution of *S*, *I*, *R* and *V* for a simulated epidemic with *β* = 0.02 and *τ*_*I*_ = 10 in random networks with *N* = 1024 and 〈*k*〉 = 10 is displayed for *α* = 0 (left) and *α* = 1 (right). We display a comparison of the final fraction of agents that are (b) infected inf_∞_ and (c) vaccinated vac_∞_ during a simulated epidemic with different values of $R_{0}$, for *α* = 0 and *α* = 1. Each point represents the median of 1000 simulation runs and the patches indicate the corresponding IQR. (d) Dependence of crossover area A on average node degree 〈*k*〉 behaves similarly in empirical social networks and model random networks. The solid line and patch shows the median and IQR of the 1000 simulated epidemics on Erdős-Rényi networks respectively. The circle and error bars represent the median and IQR of the 1000 simulated epidemic on social network of villages in southern India that have a largest connected component greater than 1000.

To gain more insight into the dynamics of the model, we simulated epidemics on Erdős-Rényi networks with *N* = 1024 and average degree 〈*k*〉 = 10, for both *α* = 0 and *α* = 1 (see Fig 3(a)–3(c)). The results are consistent with those obtained for the empirical social network. We note that similar results are obtained by increasing $R_{0}$ by changing *τ*_*I*_ instead of *β* as is done here (see S1 Fig). To see how the crossover behavior near epidemic threshold depends on the average degree 〈*k*〉 of the network, we simulated the epidemic on Erdős-Rényi networks having different average degree and on empirical social networks from the dataset of Ref. [55] whose largest connected component (LCC) size is greater than 1000. We calculated the area A enclosed between the two vac_∞_ vs $R_{0}$ curves for *α* = 0 and *α* = 1. In Fig 3(d), we have shown how this area decreases with an increase in the value of 〈*k*〉. This indicates that this intriguing behavior is dependent on the average degree of network.

**Fig 4:**
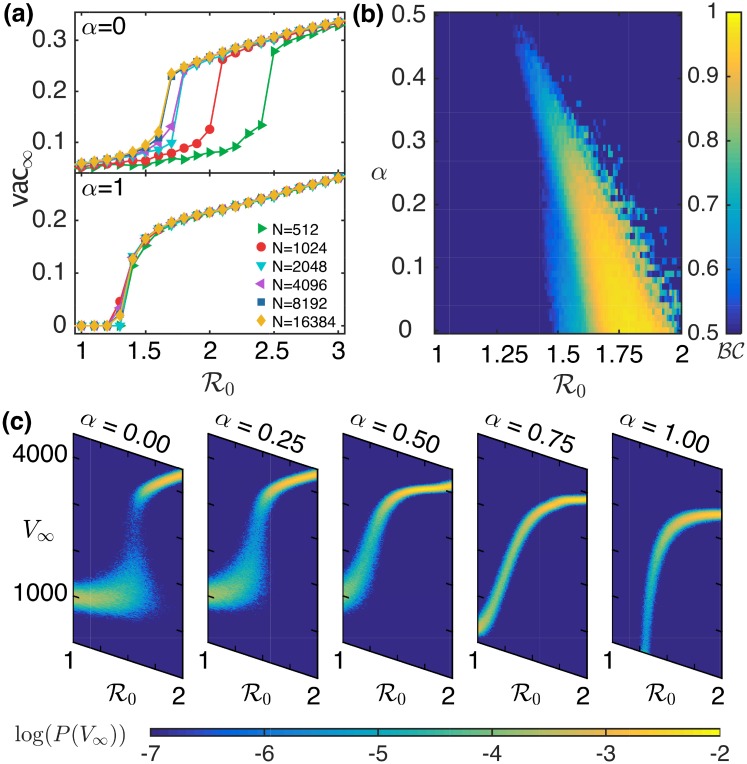
Use of local or global information by agents can qualitatively alter the collective vaccination outcome to epidemics. (a) Assessing the dependence of vac_∞_ in a network having average node degree 〈*k*〉 = 10 on population size *N*. The results are shown for *α* = 0 (top) and *α* = 1 (bottom). (b) Bimodality coefficient BC for the probability distribution of *V*_∞_ calculated over 2000 trials for Erdős-Rényi networks with *N* = 16384 and 〈*k*〉 = 10, and shown over the range of values of $R_{0}$ and *α*. (c) Probability distribution of *V*_∞_ as a function of $R_{0}$, calculated over 2000 trials, and shown for different values of *α*.

In order to examine how the results are affected by the size of the population being considered, we display the dependence of vac_∞_ on *N* for *α* = 0 (top) and *α* = 1 (bottom) in Fig 4(a). We observe that the change in the values of inf_∞_ and vac_∞_ with respect to $R_{0}$ show similar behavior on increasing the size *N* of the network. The vac_∞_ versus $R_{0}$ curves for these two different values of *α* show two different kinds of behavior, on increasing the system size. To investigate this change in behavior, we looked into the probability distribution of the final number of vaccinated agents *V*_∞_ calculated over 2000 trials. We found that for *α* = 1 this distribution is unimodal for all values of $R_{0}$, whereas for *α* = 0 a bimodal distribution is observed for some values of $R_{0}$, i.e. the probability distribution has peaks at two different locations. To identify where this behavior changes in the $\left(R_{0},\alpha \right)$ parameter space, we characterize the bimodal nature of the probability distribution of *V*_∞_ by calculating the bimodality coefficient [58]:

$$\begin{array}{c} BC=\frac{m_{3}^{2}+1}{m_{4}+3\frac{{\left(n-1\right)}^{2}}{\left(n-2)(n-3\right)}} \end{array}$$

where, *n* represents the sample size, *m*_3_ and *m*_4_ refer to the skewness and kurtosis of the distribution, respectively. A value of BC greater than 5/9 suggests that the distribution is bimodal. Our computational study indicates that the probability distribution of *V*_∞_ is bimodal for values of *α* < 0.5 [shown in Fig 4(b)]. This can be observed from Fig 4(c), which shows how the probability distribution of *V*_∞_ changes on increasing the value of *α* from 0 to 1. This can be a potential signature of a subcritical (discontinuous) transition for local information and a supercritical (continuous) transition for global information.

## Discussion

Vaccine hesitancy typically rises with decreasing disease incidence as a consequence of reduced risk perception among individuals of contracting the disease. Understanding the mechanisms driving such behavior is important as it can reverse the success of any immunization program close to achieving the eradication of a disease [59]. We utilize the framework of game theory to investigate vaccine uptake behavior, as it provides an intuitive description for the action of rational agents, i.e. in absence of any social or religious bias against decision to get vaccinated. In contrast to previous approaches, we simulate the spread of an infectious disease on a social network, where each agent can, at every time step, decide whether to get vaccinated. The decision-process of each agent is modelled by a game, in which the payoffs for different actions vary over time as the epidemic progress and the immunization status of the neighboring agents change. Each agent plays against a hypothetical opponent who shares the same neighborhood and thus has identical information, imposing symmetry on the payoff matrix. We examined whether information about an epidemic outbreak at the local or global level can lead to the emergence of voluntary vaccine uptake behavior in a population of agents that are aware of the benefits of free-riding on the immunity of their peers. In particular, we focused on how spatio-temporal heterogeneity in individuals’ vaccine uptake decisions can affect the overall vaccine coverage at the population level, and consequently determine the fate of an epidemic outbreak. We would like to stress that this heterogeneity is both in terms of the information an individual receives from the network neighborhood, as well as, the response based on her individual risk perception [60].

We observe that a defining factor for efficient disease control through voluntary vaccination is the source of information. Faster and more efficient vaccine coverage is observed for the case when individuals assess their risk of catching infection based on the prevalence in the local social network neighborhood, as opposed to that in the whole population of their social network. Compared to the size of the entire population, the number of cases that are reported in the initial phase of an epidemic are fairly low, and therefore an individual who only has access to the global prevalence information may not perceive the disease to be a serious threat. Consequently, the perception of risk in contracting the disease takes some time to become significant enough to incite vaccine uptake among individuals. However, by the time global prevalence becomes high enough so that the perceived risk of infection outweighs the cost of vaccination, the epidemic will have already affected a large fraction of the population. We find that this delay in the emergence of vaccination behavior can sometimes manifest as a large final size of the epidemic despite high vaccine coverage. On the other hand, the presence of disease in an agent’s neighborhood increases the risk of infection even at the early stage of an epidemic, and thus leads to an immediate increase in vaccine uptake. This not only increases the total vaccine coverage but also reduces the burden of disease. An intriguing observation in the case of agents using local information is that the emergence of voluntary vaccination results in bimodal distributions of the final epidemic size and vaccine coverage for diseases with $R_{0}\approx 1$. This behavior, observed close to the epidemic threshold, can be attributed to competition between the two possible final outcomes for the state of an initially susceptible individual, namely to get vaccinated or to get infected.

Previous game theory based models of vaccination during epidemic outbreaks have considered the effect of strategic decision-making in well-mixed populations where all individuals have the same risk assessment [19, 20]. In contrast, our model captures the impact of inhomogeneous risk and benefit perception at the individual level, which gives rise to spatio-temporally diverse games and hence different Nash equilibria across the population. Consequently, the whole population would never converge to a state in which every agent has the same strategy, unless the disease is completely eradicated. This also rules out the possibility that the strategic decision to vaccinate will disappear from the population with time, unlike in models that utilize imitation game dynamics to describe vaccination behavior. Indeed such models suggest that the persistence of high vaccine coverage can only be ensured by incentivizing vaccine distribution [61]. Our findings show that the model presented here provides a complementary mechanism for the emergence of voluntary vaccination. This arises as a response to the potential threat of an epidemic outbreak if each agent utilizes the information available to them and makes a rational decision whether getting vaccinated might be beneficial to her or not.

One of the key assumptions that underpins our approach is that agents are well-informed and make rational decisions based on the information available to them. In reality, the conditions under which individuals make vaccination decisions may, of course, deviate from this assumption. However, the rational agent framework, where individuals take decisions purely based on self-interest, provides a benchmark for investigating voluntary vaccination behavior. This can be then extended to include, for example, the effect of personal beliefs and peer influence [52], which can result in anti-vaccine sentiments [62] or vaccine scares [63].

While we have investigated how the final size and vaccine coverage varies for diseases with different contagiousness (i.e. $R_{0}$), it is also possible to augment our model with additional parameters that capture other features such as case fatality ratio. For instance, two diseases with comparable $R_{0}$, such as Ebola and Influenza, and thus similar transmission rate and vaccination costs, could result in different coverages, based on the subjective perception of how harmful (or severe) a disease is. The dynamics of disease progression may also be modified by including additional stages, for instance to account for appreciably long infection periods [64]. Additionally, one could also explore the consequence of differential vaccine efficacy among individuals and finite durations for the protection afforded by the vaccine. The social network on which the disease spreads has, for simplicity, been assumed to be static through the course of an epidemic. However, over time the network can indeed change by vital dynamics, i.e. through individuals dying and new ones being born. An additional source of temporal variation in the connection structure arises from the changing behavior of the agents [65] including actions taken by them in response to the epidemic, such as social distancing [66, 67].

We would like to stress that our results are independent of population size and meso-level structural details, such as the existence of modularity, but depend strongly on the degree (average number of contacts a person has) of the network. This could partly be because we are primarily considering the final outcome of the simulated epidemics, such as final epidemic size and total vaccine coverage. Another potential reason is that the strategic decision making in our model depends crucially on the neighborhood which is a micro-level detail of the social network. From a policy-making viewpoint, it is easier to estimate how many social contacts a person has on average rather than meso- and macro-level details, which widens the scope of our model and its results. We also stress on the importance of taking into account the heterogeneity in the disease status of neighbors in a social network for risk assessment when deciding whether to vaccinate. The prevalence aggregated over the whole population may sometimes result in a false perception of risk, especially if the disease is in one’s vicinity. The key outcome for public health planning is that accurate and localized reporting of disease outbreak is crucial for changing individuals’ risk perception and thereby their attitude towards vaccination, especially during the initial phase of an epidemic.

## Supporting information

S1 FigThe asymptotic fraction of infected agents inf_∞_ (a) and vaccinated agents vac_∞_ (b) in a population are shown as a function of the basic reproduction number $R_{0}$ of the simulated epidemic.As can be seen, increasing $R_{0}$ by either changing the average infectious period *τ*_*I*_ keeping the transmission rate *β* (= 0.01) constant [filled markers] or by varying *β* keeping *τ*_*I*_ (= 10) fixed [unfilled markers] result in curves that are almost identical, for both the cases of local information (*α* = 0, circles) and global information (*α* = 1, triangles).(EPS)

S2 FigProbability distributions of the ratios of the median numbers of the asymptotic fraction of infected agents inf_∞_ for *α* = 0 and *α* = 1 (a), and of the ratios of the median numbers of the asymptotic fraction of vaccinated agents vac_∞_ for *α* = 0 and *α* = 1 (b), shown as a function of the basic reproduction number $R_{0}$ of the simulated epidemic.To obtain the distributions, we randomly sampled 10^5^ parameter sets where each individual parameter value was chosen from *U*(0, 1) (uniform distribution bounded in the unit interval) from which only 127 sets were found to satisfy all the inequality relations [see Eq (1) in the main text]. We have performed 100 trials starting from randomly chosen initial conditions with each of these 127 parameter sets and obtained the median of the final fractions of infected and vaccinated agents for each of these sets. We have done this for both the extreme case scenarios, viz., exclusively local information (*α* = 0) and exclusively global information (*α* = 1), for a range of values of the basic reproduction number $R_{0}$. As expected from the results reported in the main text for inf_∞_, the ratio ${\text{inf}}_{\infty }^{1}/{\text{inf}}_{\infty }^{0}$ is always greater than unity for all values of $R_{0}$. The black squares represent median values of the distribution and a blue line, indicating a value of unity, is shown for reference.(EPS)

S3 FigSimulation results for the co-evolution of epidemic spreading and vaccine uptake behaviour for *α* in the range (0, 1).(a) A sample time series showing the evolution of *S*, *I*, *R* and *V* for a simulated epidemic with *β* = 0.02 and *τ*_*I*_ = 10 for *α* = 0.5 on a social network of villages in southern India (left) and Erdős-Rényi network (right). A comparison of the final fraction of agents infected inf_∞_ and vaccinated vac_∞_ during a simulated epidemic on a social network of villages in southern India (b and c) and Erdős-Rényi network (d and e) respectively, with different values of $R_{0}$, for *α* in the range (0, 1).(EPS)

S1 Text Pseudocode outline of the simulation algorithm. (PDF)
