# Supplementary material for: Epidemic prevalence information on social networks can mediate emergent collective outcomes in voluntary vaccine schemes
Source: PLoS Comput Biol. 2019 May 23;15(5):e1006977. doi: 10.1371/journal.pcbi.1006977 (PMC6532839; doi:10.1371/journal.pcbi.1006977)
Supplement: S1 Text — (PDF) [file pcbi.1006977.s004.pdf]

Pseudocode outline of the simulation algorithm:

input contact network G, transmission rate β, average infectious period τ_I, number of initially infected nodes I_0, payoff parameters a, b, c, d, e, f, g, h.

initialize state of all nodes as susceptible

draw I_0 nodes at random and set their state to infected

compute the no. of nodes with states susceptible (S), infected (I), recovered (R), vaccinated (V)

while I not equal to 0

- **compute** f_p and f_i for each agent
- **compute** $U_\mathrm{nv}, U_\mathrm{vv}, U_\mathrm{vn}, U_\mathrm{nn}$ for each agent
- **label** all possible events $\big[$infection ($S \to I$), recovery($I \to R$), vaccination($S \to V$)$\big]$ that can take place in the current round
- **compute** the propensities for each event: $\mathrm{P}(S \to I)$, $\mathrm{P}(I \to R)$, $\mathrm{P}(S \to V)$
- **call** random number generator
- **compute** the time interval δt between the current and the subsequent event
- **call** random number generator
- **determine** the next event E based on propensities
- **update** $t \to t + \delta t$
- **perform** the event E
- **update** the no. of nodes with states S, I, R, V

output cumulative number of infected and vaccinated nodes in the network.
